# Unfairness toward rural beneficiaries in Medicare's hierarchical conditions categories score

**DOI:** 10.1093/haschl/qxaf167

**Published:** 2025-09-23

**Authors:** Ravi B Parikh, Kristin A Linn, Junning Liang, Sae-Hwan Park, Torrey Shirk, Deborah S Cousins, Caleb Hearn, Matthew Maciejewski, Amol S Navathe

**Affiliations:** Winship Cancer Institute, Emory University, Emory Midtown, Atlanta, GA 30307, United States; The Parity Center, Perelman School of Medicine, University of Pennsylvania, Philadelphia, PA 19104, United States; The Parity Center, Perelman School of Medicine, University of Pennsylvania, Philadelphia, PA 19104, United States; The Parity Center, Perelman School of Medicine, University of Pennsylvania, Philadelphia, PA 19104, United States; The Parity Center, Perelman School of Medicine, University of Pennsylvania, Philadelphia, PA 19104, United States; The Parity Center, Perelman School of Medicine, University of Pennsylvania, Philadelphia, PA 19104, United States; The Parity Center, Perelman School of Medicine, University of Pennsylvania, Philadelphia, PA 19104, United States; The Parity Center, Perelman School of Medicine, University of Pennsylvania, Philadelphia, PA 19104, United States; Department of Population Health Sciences, Duke University, Durham, NC 27701, United States; Duke-Margolis Institute for Health Policy, Duke University, Durham, NC 27701, United States; Center of Innovation to Accelerate Discovery & Practice Transformation, Durham VA Medical Center, Durham, NC 27701, United States; The Parity Center, Perelman School of Medicine, University of Pennsylvania, Philadelphia, PA 19104, United States

**Keywords:** Medicare, risk adjustment, health policy, payment reform, rural health

## Abstract

Risk adjustment is used in healthcare payment to mitigate the payer incentive to select for healthier populations and to improve fairness of quality assessment. The Centers for Medicare and Medicaid Services (CMS) has used a spending-based metric, the CMS Hierarchical Condition Category (HCC) score, to determine risk. However, the HCC score is potentially confounded by access and utilization differences, which are related to income and rurality. In this study, we investigate how related HCC scores are to mortality, a more objective indicator of clinical risk state, and whether that relationship differs between rural and urban populations. We examined calibration of the HCC spending model by calculating the predicted-to-observed spending ratio within deciles of the HCC score. We then compared urban and rural beneficiaries’ clinical risk by comparing observed mortality rates within deciles. Our results demonstrate that the HCC model underpredicts mortality, while overpredicting spending, for rural beneficiaries. In contrast, it is well-calibrated for urban beneficiaries. These findings suggest that risk models based on HCCs may systematically disadvantage rural beneficiaries because HCC-based risk-adjusted spending may not fully account for baseline clinical risk.

## Introduction

Risk adjustment modifies healthcare payments based on individuals' clinical risk factors to ensure reimbursement and quality measures reflect the resources needed to treat patients with differing levels of clinical need. Risk adjustment is used to allocate funds for Medicare Advantage (MA) plans and appropriately account for patient comorbidities when payers reimburse hospitals and physicians, mitigating adverse selection.^1,2^ The primary risk adjustment methodology used by the Centers for Medicare and Medicaid Services (CMS) is the CMS Hierarchical Condition Category (HCC) score, which predicts individuals' future 1-year spending using demographic factors and HCCs—hierarchical groupings of diagnosis codes.^3^ HCCs and the HCC score are used to risk-adjust spending and/or quality metrics for at least 65 million Americans in MA plans, Accountable Care Organizations (ACOs), and Affordable Care Act health exchanges.^4-6^

While intended to adjust for clinical risk, the HCC system may fall short of its goals in 2 ways. First, the HCC score is derived from a spending prediction model. Spending may be a problematic outcome for a risk prediction model because, compared to more privileged populations with similar levels of clinical risk, some marginalized populations are more likely to experience barriers to accessing and utilizing care resources.^7,8^ Second, the HCC categorizations are based on diagnosis codes only. Certain groups, by virtue of lower access to primary or acute care, may be less likely to have a comorbidity coded as a diagnosis code.

One group with disproportionately lower utilization of health services is the over 60 million Americans living in rural areas.^9^ Compared to urban-dwelling beneficiaries, rural-dwelling Medicare beneficiaries may have worse clinical outcomes (burden of chronic disease and disability and age-adjusted mortality) and face disproportionately higher access barriers (lack of primary care clinicians, transportation barriers, and hospital and facility closures).^10-18^ Despite recent CMS demonstration projects to improve rural health delivery,^19,20^ spending prediction models underly risk adjustment for these programs and routine administration of Medicare benefits, creating an avenue for disparities to be perpetuated. It is important to note that Medicare Advantage base payment rates are set at the county level and reflect historical fee-for-service (FFS) spending patterns in each county, which means that existing geographic disparities in healthcare utilization are already partially embedded in the payment system before HCC risk adjustment is applied.

As the CMS-HCC is meant to serve as a proxy for clinical risk, comparing the HCC to a more objective measure of clinical risk may reveal the magnitude and mechanisms of unfairness against rural beneficiaries. Mortality can serve as an objective clinical measure, as it is uniformly measured across the Medicare population, not subjective to utilization-related issues in its measurement, and may have a more direct relationship with diagnosis code-based HCC categories. The financial implications of HCC-based risk adjustment miscalibrations are substantial: If rural beneficiaries receive systematically lower HCC scores than their urban counterparts with similar actual clinical risk, this translates directly into reduced payments to rural providers and Medicare Advantage plans serving these communities, potentially exacerbating existing healthcare access disparities in already underserved areas. In this study, we hypothesized that the HCC system may systematically disadvantage rural-dwelling beneficiaries by relying on spending patterns to predict risk, likely underestimating the risk of mortality.

## Methods

This study was approved by the (institution omitted) Institutional Review Board with a waiver of informed consent because only historical data with minimal risk of harm were used. This study followed the Strengthening the Reporting of Observational Studies in Epidemiology (STROBE) reporting guideline.

### Data collection

We used a nationally representative 5 million random sample (*n* = 4 170 277) of traditional (fee-for-service) Medicare beneficiaries. We included dually- and non-dually eligible community-dwelling individuals. Part A and B medical carrier, outpatient, inpatient, and skilled nursing facility claims were included since they are used by CMS to train the HCC model. Hospice and durable medical equipment claims were unavailable.

### Study periods and sample

**Figure 1.**
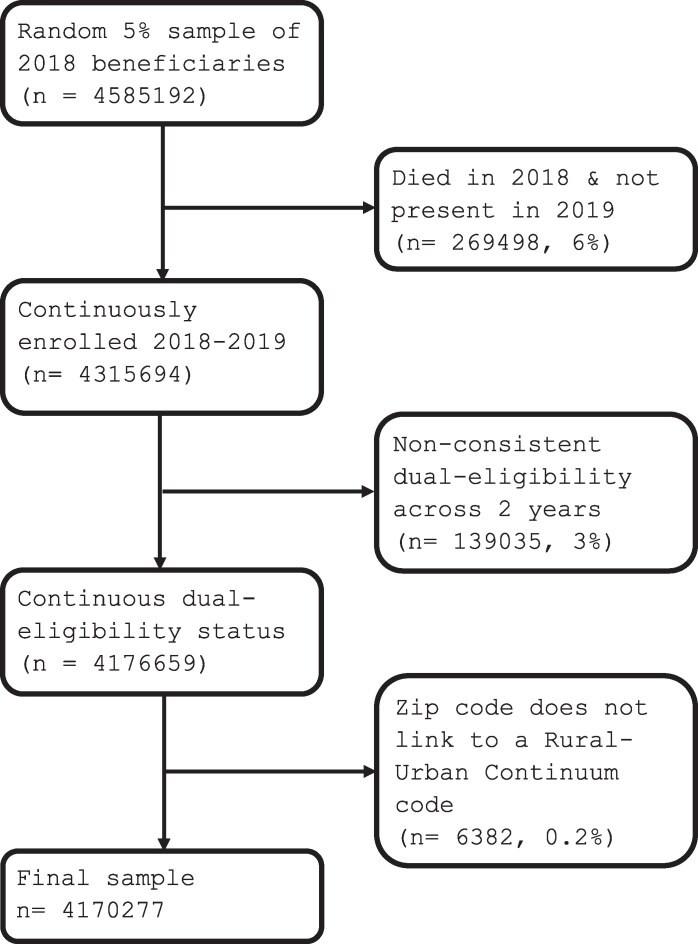
Study sample. Source/Notes: Author's analysis of data from the Medicare fee-for-service Master Beneficiary Summary File (MBSF), 2018-2019.

Our study period spanned dates from January 1, 2018, through December 31, 2019. Our baseline period included dates from January 1, 2018, through December 31, 2018, and the follow-up period spanned January 1, 2019, through December 31, 2019. Models used demographic variables and diagnosis codes recorded in 2018 to predict total medical cost and mortality in 2019. The study sample included Medicare fee-for-service beneficiaries who met eligibility criteria by being aged (age ≥ 65 years) or disabled, and did not have end-stage renal disease. We excluded patients with end-stage renal disease because CMS uses a separate HCC score for this population. This segment is the largest in Medicare, making up 99% of all traditional Medicare (TM) beneficiaries. Dually eligible beneficiaries, who receive both Medicare and Medicaid benefits, were included. We excluded beneficiaries with insurance coverage through Medicare Advantage, as well as beneficiaries who died in 2018 or did not have continuous enrollment in a primary Medicare fee-for-service period throughout the study period. Note that individuals who died in 2019 were retained. See Figure 1 for the cohort eligibility schema.

We identified rural and urban beneficiaries by using definitions from the Health Resources and Services Administration. Specifically, in our primary analysis, we followed 2013 Office of Management and Budget (OMB) delineations which define counties in nonmetro areas with 2013 Rural–Urban Continuum Codes (RUCCs) 4-9 as rural. Counties with 2013 RUCCs 1-3 were classified as urban (see Table S1 for details).

### Outcomes and covariates

The CMS-HCC v24 score is a weighted sum of 86 categories corresponding to recorded diagnosis codes (see Supplementary material for further description). To predict the outcome of 1-year spending in calendar year 2019, we used the same variables used in CMS-HCC v24 (age, sex, and the HCC categories) as covariates. All covariates were measured between January 1, 2018, and December 31, 2018. We used published weights (coefficients) from HCC software V24 (version year 2021), which aligns with years 2018-2019 as per CMS documentation.^21^ HCC scores consisted of 3 components:

1. An expert-curated hierarchy of conditions (“categories”)
2. An assignment of ICD codes to the categories
3. A positive linear weight for each category and weights for age and sex groups

We followed CMS methods in normalizing the HCC score.^22^ Predicted spending was estimated by first normalizing the HCC score within each of the dual-eligibility segments such that the mean HCC score in each segment was set to 1. This normalization was achieved by dividing each beneficiary's HCC score by the mean of their respective segment. The normalized HCC score was then multiplied by the average observed spending within the corresponding segment to generate the final predicted spending estimate in dollars. To account for mortality and subsequent months of non-spend for patients who died in calendar year 2019, we calculated annualized spending by dividing the deceased patient's total 2019 spending by the number of months of enrollment and multiplying the result by 12.

### Statistical analysis

Characteristics between urban and rural beneficiaries were compared using standardized differences of means and proportions. Race was defined using the Medicare Research Triangle Institute (RTI) Race Code from the Master Beneficiary Summary File (MBSF). Beneficiaries were categorized into 3 groups: White (RTI code = 1), Black or African American (RTI code = 2), and other (all remaining RTI codes combined into a single category). We calculated all results for non-dually eligible and dually eligible beneficiaries separately. All primary analysis models were fit at the individual beneficiary level.

We first assessed calibration of the HCC spending model by binning HCC-predicted spending into deciles and plotting observed spending within each decile bin. To compare calibration between urban and rural beneficiaries, we calculated predictive ratios, the ratio of predicted-to-observed spending, within decile bins of predicted spending risk. We included pointwise 95% confidence intervals for observed spending at each decile bin of predicted spending.

To compare clinical risk between urban and rural beneficiaries at common levels of spending risk, we descriptively compared observed mortality rates in each group at decile bins of predicted spending risk. To predict clinical risk, we stratified beneficiaries into the 6 CMS-defined community groups based on dual eligibility and original Medicare entitlement reason and then fit separate logistic regression models for each group using the same demographic and HCC covariates as the CMS-HCC V24 spending model. The model was developed by splitting the dataset into 60% training and 40% testing subsets, with performance evaluated on the test set. The primary outcome of the clinical prediction model was mortality (deceased or alive), obtained using the Medicare Master Beneficiary Summary File to ascertain date of death. We combined predictions across all models weighted by each group's population share. Model coefficients and performance metrics are provided in the Supplementary material.

To compare model goodness-of-fit across urban and rural subgroups, we calculated excess mortality and excess spending by taking the difference between observed and model-predicted values for each individual in the test set. These residual values represent over- and underprediction of mortality and spending after adjusting for age, sex, and HCCs. We then plotted excess mortality and spending by stratifying the test set into deciles (every 10th percentile) of predicted mortality risk separately for urban and rural subgroups.

Given the large sample size and focus on overall calibration trends rather than hypothesis testing, we did not adjust for multiple comparisons or additional confounding variables beyond those included in the HCC model.

### Sensitivity analyses

We accounted for differing definitions of rurality by repeating our analyses with rurality defined using a conservative definition of Rural–Urban Continuum Codes 7-9 and Core-Based Statistical Area = Rural.

To account for potential differences in pricing between rural vs urban areas, we conducted another sensitivity analysis using normalized spending by claim type. To facilitate consistent comparisons of healthcare utilization across providers, Medicare claims were subjected to a standardized costing method that was adjusted to 2019 dollars. Diagnosis-related group (DRG) weights were used to determine inpatient costs. Healthcare Common Procedure Coding System (HCPCS) codes and fee schedules, which were modified for facility type and inflation, were used to pay outpatients and providers. Using their corresponding classification schemes and base payments, post-acute care costs (SNF, IRF, and HHA) were standardized. This method eliminates regional and provider-specific variances to guarantee comparability.

Finally, to account for potential differences in HCC score accuracy between our sample and the overall Medicare population, we conducted another sensitivity analysis in which the HCC spending model was retrained using ordinary least squares regression on our 2018 cohort. The retrained model used beneficiary-level annualized observed spending as the outcome and the 86 HCCs, age, and sex as covariates.

### Limitations

Our study has several limitations. Our inability to observe post-processing adjustments of HCC scores introduces uncertainty regarding their downstream policy applications. Furthermore, our results may not generalize beyond the 2018-2019 period and traditional Medicare beneficiaries, potentially differing in Medicare Advantage or other populations. We also do not observe diagnoses collected through health risk assessments, although we expect this would only widen the gaps observed since rural beneficiaries are less likely to receive home nurse visits for this purpose. Nonetheless, our findings align with prior literature and provide a robust basis for addressing inequities in risk adjustment. Finally, while our sample size for certain subpopulations such as rural dual eligibles (*n* ≈ 150 000) represents a substantial number of beneficiaries from which to draw population-level inferences, we acknowledge that precision for subgroup analyses may be somewhat limited.

## Results

There were 4 170 277 beneficiaries (Table 1) in the sample (624 649 [14.98%] dually eligible; 3 545 628 [85.02%] non-dually eligible; mean [SD] age 70.59 [11.41], 2 214 762 [53.11%] female, 377 610 [9.05%] Black, 3 442 109 [82.54%] White, 350 558 [8.41%] individuals of other race/ethnicity). There were 874 847 rural beneficiaries (mean [SD] age 70.17 [11.54], 454 975 [52%] female, 419 872 [48%] male, 50 635 [5.8%] Black, 782 950 [89.5%] White, 41 262 [4.7%] individuals of other race/ethnicity) and 3 295 430 urban beneficiaries (mean [SD] age 70.69 [11.37], 1 759 787 [53.40%] female, 1 535 643 [46.60%] male, 326 975 [9.9%] Black, 2 659 159 [80.7%] White, 309 296 [9.4%] individuals of other race/ethnicity). One-year mean spending was $9060.08 for the whole sample, with rural-dwelling beneficiaries averaging $8640.82 (5.8% lower than the overall mean) and urban-dwelling beneficiaries averaging $9171.38 (1.2% higher than the overall mean).

**Table 1.** Demographic characteristics.

	Total (*n* = 4 170 277)	Urban (*n* = 3 295 430)	Rural (*n* = 874 847)
Age, mean (SD)	70.59 (11.41)	70.69 (11.37)	70.17 (11.54)
Sex, *n* (%)			
Male	1 955 515 (46.89)	1 535 643 (46.60)	419 872 (48.00)
Female	2 214 762 (53.11)	1 759 787 (53.40)	454 975 (52.00)
Race, *n* (%)			
Black	377 610 (9.05)	326 975 (9.9)	50 635 (5.8)
White	3 442 109 (82.54)	2 659 159 (80.7)	782 950 (89.5)
Other	350 558 (8.41)	309 296 (9.4)	41 262 (4.7)
Geographic location, *n* (%)			
Midwest	923 647 (22.15)	646 335 (19.61)	277 312 (31.7)
Northeast	776 799 (18.63)	690 083 (20.94)	86 716 (9.91)
South	1 633 883 (39.18)	1 261 648 (38.29)	372 235 (42.55)
West	835 948 (20.04)	697 364 (21.16)	138 584 (15.84)
Beneficiary status, *n* (%)			
Full-dual aged	254 652 (6.11)	202 944 (6.16)	51 708 (5.91)
Full-dual disabled	227 666 (5.46)	173 040 (5.25)	54 626 (6.24)
Non-dual aged	3 215 692 (77.11)	2 568 463 (77.94)	647 229 (73.98)
Non-dual disabled	329 936 (7.91)	255 064 (7.74)	74 872 (8.56)
Partial-dual aged	76 027 (1.82)	50 503 (1.53)	25 524 (2.92)
Partial-dual disabled	66 304 (1.59)	45 416 (1.38)	20 888 (2.39)
Mortality, *n* (%)	162 513 (3.9)	125 342 (3.8)	37 171 (4.25)
Average HCC score	1.19 (1.28)	1.19 (1.29)	1.17 (1.23)
Normalized average HCC score	1.00 (1.03)	1.00 (1.04)	0.98 (0.98)

Source: Author's analysis of data from the Medicare fee-for-service Master Beneficiary Summary File (MBSF) and CMS risk-adjusted HCC scores, 2018-2019.

In operations, CMS normalizes HCC scores to have a mean of 1.0. The fact that average HCC scores are greater than 1 in this analysis is due to 3 factors: (1) Scores in this analysis represent non-normalized scores; (2) CMS normalizes scores within each specific segment of the HCC score, whereas various segments are aggregated together in this analysis; and (3) the scores in this analysis represent a different year from which CMS-HCC v24 was trained.

### Calibration of the HCC spending model

**Figure 2.**
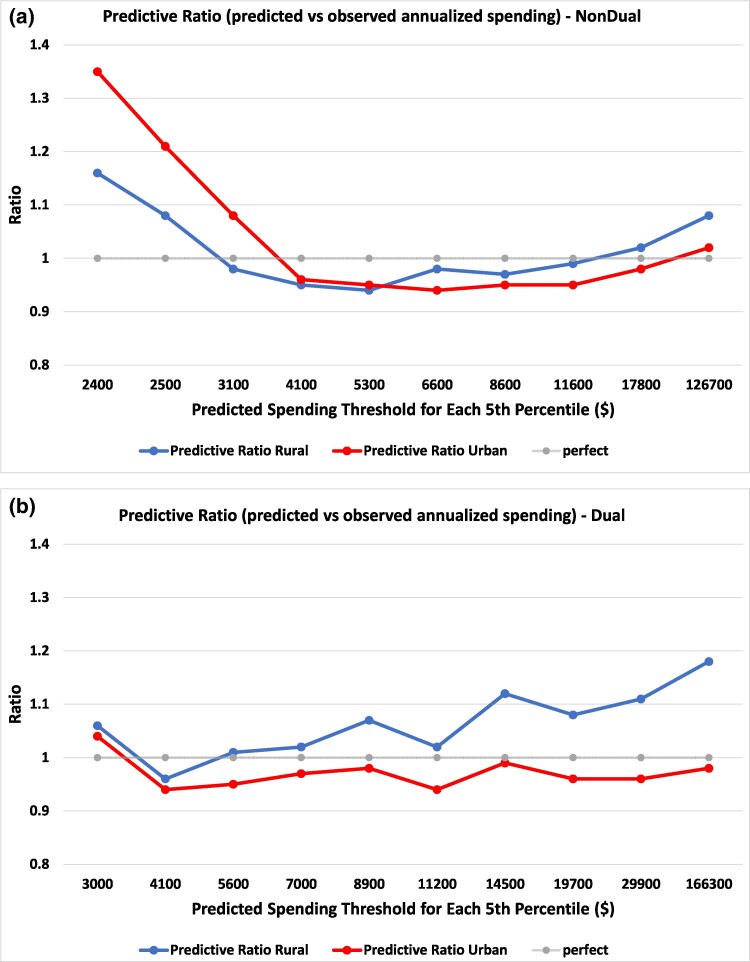
HCC-predicted spending vs observed annualized spending. Source/Notes: Author's analysis of data from the Medicare fee-for-service Master Beneficiary Summary File (MBSF), MedPAR claims, outpatient claims, home health claims, carrier claims, and CMS risk-adjusted HCC scores, 2018-2019. This figure represents predictive ratios of overall spending for non-dually eligible beneficiaries (a) and dually eligible beneficiaries (b). The *x*-axis represents predicted spending thresholds at every 10th percentile, obtained by binning the data into equal-sized groups and assigning the predicted spending amount at each percentile cutoff. The *y*-axis represents the predictive ratio, calculated as the mean predicted spending divided by the mean observed spending within each bin.

Among non-dually eligible beneficiaries, the HCC spending model systematically overpredicted spending across most risk bins for rural beneficiaries, while it was well-calibrated for urban beneficiaries (Figure 2a). This finding was magnified among dually eligible beneficiaries (Figure 2b). Among non-dually eligible beneficiaries, the HCC score overpredicted spending for rural beneficiaries (Figure S1A). In the overall sample, mean predicted vs observed spending was $8112 vs $7924 (predictive ratio 1.02, mean difference $188). In the top 50% of the predicted spending distribution, mean predicted vs observed spending was $12 200 vs $11 866 (predictive ratio 1.03, mean difference $334).

Among dually eligible beneficiaries, overprediction of spending was more marked for rural-dwelling individuals (Figure S1B). In the overall sample, mean predicted vs observed spending was $12 810 vs $11 599 (predictive ratio 1.10, mean difference $1211). Results were consistent when we repeated the analysis using the retrained HCC score (Figures S2 and S3).

### Calibration of the mortality model

One-year mortality rates were 4.25% for rural-dwelling beneficiaries and 3.80% for urban-dwelling beneficiaries. Mortality predictions based on HCCs were not well-calibrated for rural beneficiaries, with HCCs underpredicting mortality, as indicated by an observed/expected (O/E) ratio of 1.17. For urban beneficiaries, the O/E ratio was 0.96.

### Observed mortality by spending risk

**Figure 3.**
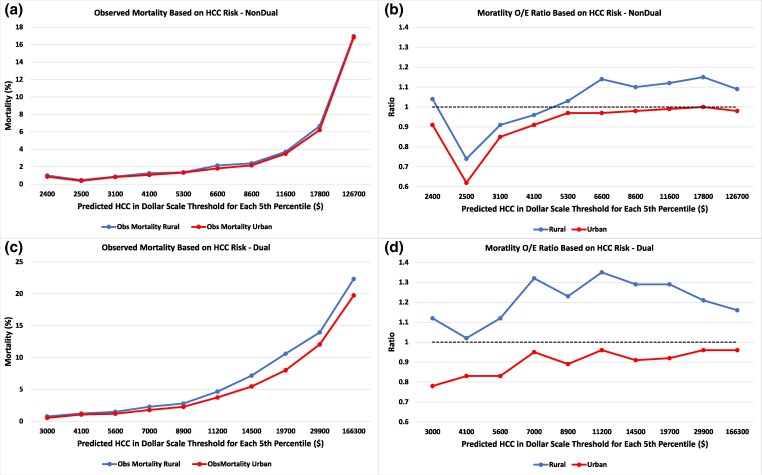
Observed vs predicted mortality among non-dually insured and dually insured beneficiaries. Source/Notes: Author's analysis of data from the Medicare fee-for-service Master Beneficiary Summary File (MBSF) and CMS risk-adjusted HCC scores, 2018-2019. Note: represents observed vs predicted mortality for non-dual-eligible beneficiaries (a and b) and dual-eligible beneficiaries (c and d). a and b show observed mortality across predicted spending thresholds. The *x*-axis represents the predicted spending threshold at every 10th percentile, and the *y*-axis shows the mean observed mortality within each bin. b and d present the mortality observed-to-expected (O/E). The *x*-axis represents the predicted spending threshold at every fifth percentile, and the *y*-axis shows the ratio of observed to expected mortality within each bin.

Across all deciles of HCC-predicted spending, observed mortality exceeded predicted mortality for non-dually eligible rural beneficiaries (Figure 3a and b; mean predicted vs observed mortality: 3.41% vs 3.69%, observed/expected ratio 1.04). In contrast, predicted mortality exceeded observed mortality for urban beneficiaries (3.61% vs 3.50%, observed/expected ratio 0.91). These findings were more pronounced for beneficiaries who were dual-eligible (Figure 3c and d): rural (5.53% vs 6.73%, observed/expected ratio 1.22) and urban (5.96% vs 5.59%, observed/expected ratio 0.94).

### Excess mortality

Mean excess mortality was greater for rural beneficiaries regardless of duality status (Figure S2A and B). Excess mortality was 0.72% with the 95% confidence interval (0.10%, 1.35%) for non-dual rural beneficiaries and 0.03% (−0.30%, 0.36%) for non-dual urban beneficiaries in the top 50% of the predicted spending distribution, corresponding to 144 191 and 564 824 individuals in our sample, respectively. For dual beneficiaries in the top 50% of the predicted spending distribution, excess mortality was 2.34% (0.73%, 3.94%) for rural beneficiaries and −0.54% (−1.53%, 0.44%) for urban beneficiaries, corresponding to 30 601 and 94 268 individuals in our sample, respectively.

### Sensitivity analyses

In sensitivity analyses using an alternative definition of rurality, the results were consistent with the primary analysis (Figure S6).

In a sensitivity analysis where we retrained the spending model using standardized spending, spending was less miscalibrated compared to the original HCC spending model. However, the relative overprediction of spending for rural beneficiaries was consistent with primary results (Figure S6).

## Discussion

We demonstrated that Medicare's Hierarchical Condition Category (HCC) scoring system may disadvantage rural beneficiaries in ways that could impact both payment equity and health outcomes. By comparing HCC-predicted spending risk with mortality risk, an objective measure of clinical risk, we find that spending is a poor proxy for an important measure of clinical risk (ie, mortality risk) among rural beneficiaries. Previous studies^23^ have documented lower HCC scores in rural areas and examined coding patterns for specific conditions. Our analysis uniquely correlates HCC-predicted spending with actual mortality risk to demonstrate that spending-based models systematically underestimate clinical risk for rural beneficiaries, revealing a fundamental calibration issue with the HCC for rural individuals. In other words, even if spending is overpredicted for rural beneficiaries based on HCCs, HCCs are undercalibrated for predicting a more clinically salient measure of risk (eg, mortality). Thus, using an alternative, clinically relevant risk adjustment tool may lead to increased risk-adjusted payments to rural beneficiaries. The overprediction of spending coupled with the underprediction of mortality for rural beneficiaries highlights potential limitations of using spending-based models as the basis for risk adjustment.

There are several policy implications of our findings. First, the HCC system is central to the risk adjustment used in Medicare fee-for-service (FFS), Medicare Advantage (MA), and other programs. In these settings, the HCC system influences quality metrics, spending targets for Accountable Care Organizations (ACOs), and mortality prediction. Our findings suggest that rural beneficiaries and systems serving them may be systematically disadvantaged in these programs, receiving inadequate adjusted reimbursements or quality scores compared to the true clinical risk of their populations. This under-adjustment may undermine the financial sustainability of rural healthcare providers and facilities, further exacerbating healthcare access challenges. Medicare Advantage plans could also be incentivized to target rural beneficiaries who may spend less than expected based on their HCC scores. In other words, rural beneficiaries may be more profitable than non-rural beneficiaries due to their lower access, utilization, and spending despite their higher clinical risk. Such selective enrollment could perpetuate inequities, as MA strategies such as narrow networks and prior authorization may limit further access to care for rural beneficiaries. Alternatively, however, the relatively higher payments (vs comparable urban-dwelling beneficiaries) may give MA plans more resources to address the higher clinical risk. More research is required to investigate how these opposing incentives play out for rural beneficiary health outcomes. Our findings have direct implications for healthcare financing and access. When the HCC system underpredicts clinical risk for rural beneficiaries, it results in systematically lower risk-adjusted payments to providers and plans serving these populations than those if their true clinical risk was appropriately captured. This payment inequity may discourage provider participation in rural areas, limit plan offerings, and ultimately reduce access to care for the 60 million Americans living in rural communities. Furthermore, quality metrics that rely on HCC risk adjustment may unfairly penalize rural providers by inadequately accounting for their patients' true clinical complexity.

Second, the hierarchical grouping of the HCCs themselves, which are based on diagnosis codes, may contribute to disparities in score accuracy between urban and rural beneficiaries. These codes are generated through encounters with the healthcare system, often in outpatient or acute care settings. Rural beneficiaries, facing reduced access to primary and acute care, may be less likely to have diagnoses appropriately captured or have access to certain types of services that capture diagnostic codes. For example, rural-dwelling beneficiaries are less likely to receive home visits for medical social service or therapeutic visits, which may result in lower numbers of certain diagnoses captured via home visits.^24^ While we did not use home health diagnoses in calculating HCC spending scores (as CMS does not include them), the fact that rural beneficiaries have reduced access to home health services that could capture additional diagnoses further supports our argument that the HCC system may systematically undercapture the clinical complexity of rural populations. This limitation is starkly reflected in the lower number of HCCs coded at similar levels of spending risk for rural beneficiaries and underscores a defect in the foundation of risk adjustment through HCCs.

Third, prior evidence has shown that spending is a poor proxy for clinical risk among other disadvantaged populations.^7,8^ Our results suggest that such label bias extends to the HCC system with respect to rural-dwelling beneficiaries, similar to the racial and ethnic disparities previously identified in evaluations of spending prediction models. In particular, a model using HCCs to predict mortality is algorithmically unfair to rural beneficiaries, demonstrating a systematic undercalibration of mortality risk. While previous research^25^ has shown that HCC scores provide a reasonable predictive signal for mortality, our analysis reveals meaningful calibration gaps when spending model inputs are used to predict mortality. The systematic underprediction of mortality for rural beneficiaries despite reasonable overall model performance (O/E ratio 1.17 for rural vs 0.96 for urban) demonstrates that spending-based risk adjustment inherently disadvantages rural populations. This calibration disparity persists even when HCCs show moderate discriminative ability for mortality, underscoring that the issue lies not with the clinical categorizations themselves but with their weights when trained on spending rather than clinical outcomes^25^ and their ability to accurately reflect true underlying clinical risk. This suggests that spending-based risk adjustment frameworks may exacerbate existing disparities across different marginalized populations. Multiple factors—including rural-dwelling patients' lack of access to comprehensive treatment and diagnostics and greater reliance on primary over specialty care, and individuals with more complex health needs preferentially seeking care in urban areas—contribute to observed differences in spending between urban and rural beneficiaries with similar comorbidity profiles.^26,27^

Fourth, inadequate accounting for social determinants of health (SDOH) may disproportionately affect rural populations relative to urban populations. Barriers such as reduced transportation access, limited diagnostic testing, and lower quality of clinical documentation disproportionately affect rural beneficiaries. These factors result in under-coding of comorbidities and, consequently, under-adjustment of risk scores. The failure to fully capture these social factors compounds the structural inequities already faced by rural populations, leaving them at a disadvantage in risk adjustment models reliant on spending-based metrics. As these issues may impact access, this mechanism likely mechanically produces spending overpredictions in the face of clinical risk underprediction.

Fifth, Medicare Advantage enrollment is indeed lower in rural areas, partly due to limited plan availability and provider network challenges in sparsely populated regions. Our findings suggest that HCC-based payment inadequacy may contribute to this disparity by making rural markets less attractive to MA plans and providers.

Our findings inform potential strategies to mitigate urban-rural bias in HCC models. First, incorporating additional geographic or SDOH covariates into the HCC model could improve the accuracy of risk adjustment. However, area-level deprivation indices and small numbers of individual survey-based metrics (eg, financial strain, food insecurity, utility insecurity, unreliable transportation, housing insecurity, and loneliness) alone may be insufficient, as previous research among Medicare Advantage members found that incorporating these SDOH variables into the HCC score actually lowered predicted spending and failed to reduce payment inequities for rural beneficiaries.^28^ Thus, more granular individual-level SDOH metrics focusing on access to health care may be needed. Second, incorporating interaction terms between rurality and clinical risk factors may reduce the observed geographic disparities and improve the accuracy of risk adjustment. Tailoring models to rural populations, as is considered in programs like the Community Health Access and Rural Transformation (CHART) Model, would help address structural inequities and ensure fairer comparisons between urban and rural beneficiaries.^29^ Interaction terms could help adjust for nuances within rural populations, such as the compounded effects of dual eligibility. Third, collecting gold-standard diagnosis validation data from well-represented surveys or health-related questionnaires may facilitate algorithms to impute missing diagnoses and improve calibration. This approach, which does not depend on access to or use of care, aligns with efforts like CMS's Quality Payment Program^30^ which emphasizes data collection and validation to support alternative payment models. Importantly, data collection from clinical data sources such as electronic health records may be expected to have similar issues as claims with undercapture of clinical data. Enhanced data collection could mitigate the effects of underdiagnosis and ensure that rural populations are equitably represented in risk adjustment. This effort would require substantial investment but could yield long-term benefits by improving both fairness and accuracy in predictive models.

In conclusion, our study underscores the pressing need to address disparities in Medicare's risk adjustment framework to ensure equitable resource allocation for rural beneficiaries. By adopting data-driven policy solutions, CMS and other stakeholders can mitigate disparities and improve health equity for one of Medicare's most vulnerable populations.

## Supplementary Material

qxaf167_Supplementary_Data
