# Supplementary material for: Unfairness toward rural beneficiaries in Medicare's hierarchical conditions categories score
Source: Health Aff Sch. 2025 Sep 23;3(9):qxaf167. doi: 10.1093/haschl/qxaf167 (PMC12456169; doi:10.1093/haschl/qxaf167)
Supplement: qxaf167_Supplementary_Data [file qxaf167_supplementary_data.zip › Urban.Rural.Bias.HCC_Supplement.docx]

**Supplement**

**Table of Contents**

1. Appendix Exhibit 1. Spending Based on Mortality Risk
2. Supplemental Methods. Outcomes and covariates
3. Supplemental Exhibit 1. Breakdown of beneficiaries by urban-rural designation
4. Supplemental Exhibit 2A-B. Calibration plot of original HCC spending model
5. Supplemental Exhibit 3. Calibration plot using our retrained HCC spending model
6. Supplemental Exhibit 4. Predictive ratios of overall spending in retrained HCC Model.
7. Supplemental Exhibit 5A-B. Excess mortality among non-dually insured and dually-insured beneficiaries.
8. Supplemental Exhibit 6A-E: Sensitivity analyses using alternative definition of rurality.
9. Supplemental Exhibit 7A-B: Sensitivity analyses using standardized spending.

Appendix Exhibit 1

Figure A


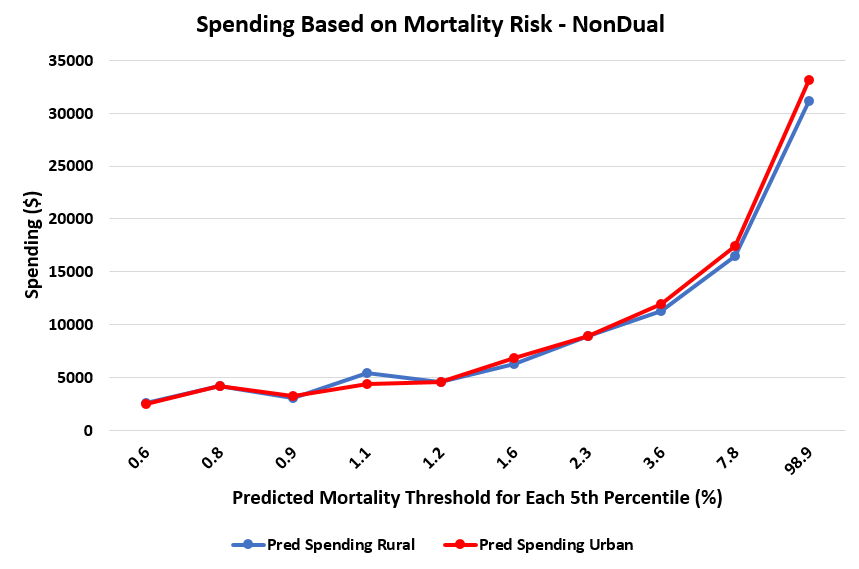


Appendix Exhibit 1

Figure B


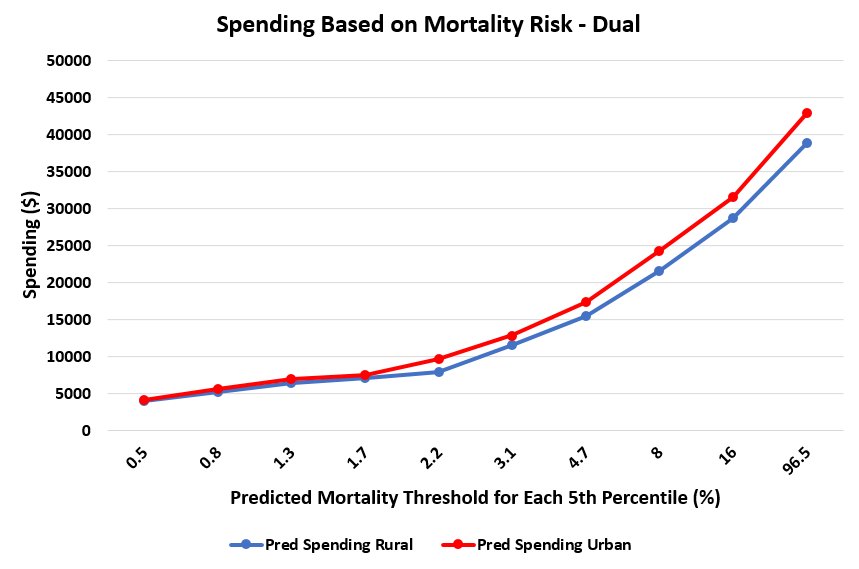


Appendix:

Appendix Exhibit 1 represents predicted spending based on mortality risk for non-dual-eligible beneficiaries (Figure A) and dual-eligible beneficiaries (Figure B). The x-axis represents predicted mortality risk at every 5th percentile threshold, and the y-axis shows the mean predicted spending amount in dollars within each bin.

Supplemental Methods:

Outcomes and covariates

86 HCC categories from V24 software

HCC1 ="HIV/AIDS "

HCC2 ="Septicemia, Sepsis, Systemic Inflammatory Response Syndrome/Shock "

HCC6 ="Opportunistic Infections "

HCC8 ="Metastatic Cancer and Acute Leukemia "

HCC9 ="Lung and Other Severe Cancers "

HCC10 ="Lymphoma and Other Cancers "

HCC11 ="Colorectal, Bladder, and Other Cancers "

HCC12 ="Breast, Prostate, and Other Cancers and Tumors "

HCC17 ="Diabetes with Acute Complications "

HCC18 ="Diabetes with Chronic Complications "

HCC19 ="Diabetes without Complication "

HCC21 ="Protein-Calorie Malnutrition "

HCC22 ="Morbid Obesity "

HCC23 ="Other Significant Endocrine and Metabolic Disorders "

HCC27 ="End-Stage Liver Disease "

HCC28 ="Cirrhosis of Liver "

HCC29 ="Chronic Hepatitis "

HCC33 ="Intestinal Obstruction/Perforation "

HCC34 ="Chronic Pancreatitis "

HCC35 ="Inflammatory Bowel Disease "

HCC39 ="Bone/Joint/Muscle Infections/Necrosis "

HCC40 ="Rheumatoid Arthritis and Inflammatory Connective Tissue Disease "

HCC46 ="Severe Hematological Disorders "

HCC47 ="Disorders of Immunity "

HCC48 ="Coagulation Defects and Other Specified Hematological Disorders "

HCC51 ="Dementia With Complications "

HCC52 ="Dementia Without Complication "

HCC54 ="Substance Use with Psychotic Complications "

HCC55 ="Substance Use Disorder, Moderate/Severe, or Substance Use with Complications "

HCC56 ="Substance Use Disorder, Mild, Except Alcohol and Cannabis "

HCC57 ="Schizophrenia "

HCC58 ="Reactive and Unspecified Psychosis "

HCC59 ="Major Depressive, Bipolar, and Paranoid Disorders "

HCC60 ="Personality Disorders "

HCC70 ="Quadriplegia "

HCC71 ="Paraplegia "

HCC72 ="Spinal Cord Disorders/Injuries "

HCC73 ="Amyotrophic Lateral Sclerosis and Other Motor Neuron Disease "

HCC74 ="Cerebral Palsy "

HCC75 ="Myasthenia Gravis/Myoneural Disorders and Guillain-Barre Syndrome/Inflammatory and Toxic Neuropathy "

HCC76 ="Muscular Dystrophy "

HCC77 ="Multiple Sclerosis "

HCC78 ="Parkinson's and Huntington's Diseases "

HCC79 ="Seizure Disorders and Convulsions "

HCC80 ="Coma, Brain Compression/Anoxic Damage "

HCC82 ="Respirator Dependence/Tracheostomy Status "

HCC83 ="Respiratory Arrest "

HCC84 ="Cardio-Respiratory Failure and Shock "

HCC85 ="Congestive Heart Failure "

HCC86 ="Acute Myocardial Infarction "

HCC87 ="Unstable Angina and Other Acute Ischemic Heart Disease "

HCC88 ="Angina Pectoris "

HCC96 ="Specified Heart Arrhythmias "

HCC99 ="Intracranial Hemorrhage "

HCC100 ="Ischemic or Unspecified Stroke "

HCC103 ="Hemiplegia/Hemiparesis "

HCC104 ="Monoplegia, Other Paralytic Syndromes "

HCC106 ="Atherosclerosis of the Extremities with Ulceration or Gangrene "

HCC107 ="Vascular Disease with Complications "

HCC108 ="Vascular Disease "

HCC110 ="Cystic Fibrosis "

HCC111 ="Chronic Obstructive Pulmonary Disease "

HCC112 ="Fibrosis of Lung and Other Chronic Lung Disorders "

HCC114 ="Aspiration and Specified Bacterial Pneumonias "

HCC115 ="Pneumococcal Pneumonia, Empyema, Lung Abscess "

HCC122 ="Proliferative Diabetic Retinopathy and Vitreous Hemorrhage "

HCC124 ="Exudative Macular Degeneration "

HCC134 ="Dialysis Status "

HCC135 ="Acute Renal Failure "

HCC136 ="Chronic Kidney Disease, Stage 5 "

HCC137 ="Chronic Kidney Disease, Severe (Stage 4) "

HCC138 ="Chronic Kidney Disease, Moderate (Stage 3) "

HCC157 ="Pressure Ulcer of Skin with Necrosis Through to Muscle, Tendon, or Bone "

HCC158 ="Pressure Ulcer of Skin with Full Thickness Skin Loss "

HCC159 ="Pressure Ulcer of Skin with Partial Thickness Skin Loss "

HCC161 ="Chronic Ulcer of Skin, Except Pressure "

HCC162 ="Severe Skin Burn or Condition "

HCC166 ="Severe Head Injury "

HCC167 ="Major Head Injury "

HCC169 ="Vertebral Fractures without Spinal Cord Injury "

HCC170 ="Hip Fracture/Dislocation "

HCC173 ="Traumatic Amputations and Complications "

HCC176 ="Complications of Specified Implanted Device or Graft "

HCC186 ="Major Organ Transplant or Replacement Status "

HCC188 ="Artificial Openings for Feeding or Elimination "

HCC189 ="Amputation Status, Lower Limb/Amputation Complications "

**Supplemental Exhibit 1. Breakdown of beneficiaries by urban-rural designation**

| Beneficiary Group | Total | RUCC 4-9 | RUCC 7-9 | CBSA-Rural |
| --- | --- | --- | --- | --- |
| Total | 4170277  (100%) | 874847 (21%) | 275909 (7%) | 868288 (21%) |
| CFA | 254652 | 51708 | 17215 | 51343 |
| CFD | 227666 | 54626 | 16360 | 54282 |
| CNA | 3215692 | 647229 | 203042 | 641959 |
| CND | 329936 | 74872 | 23342 | 74485 |
| CPA | 76027 | 25524 | 8970 | 25410 |
| CPD | 66304 | 20888 | 6980 | 20809 |

NOTE: CFA: Community – Full Benefit dual aged

CFD: Community – Full Benefit dual disabled

CNA: Community – Non-dual aged

CND: Community – Non-dual disabled

CPA: Community – Partial Benefit dual aged

CPD: Community – Partial Benefit dual disabled

**Supplemental Exhibit 2**: **Calibration plot of original HCC spending Model.**Supplemental Exhibit 2A


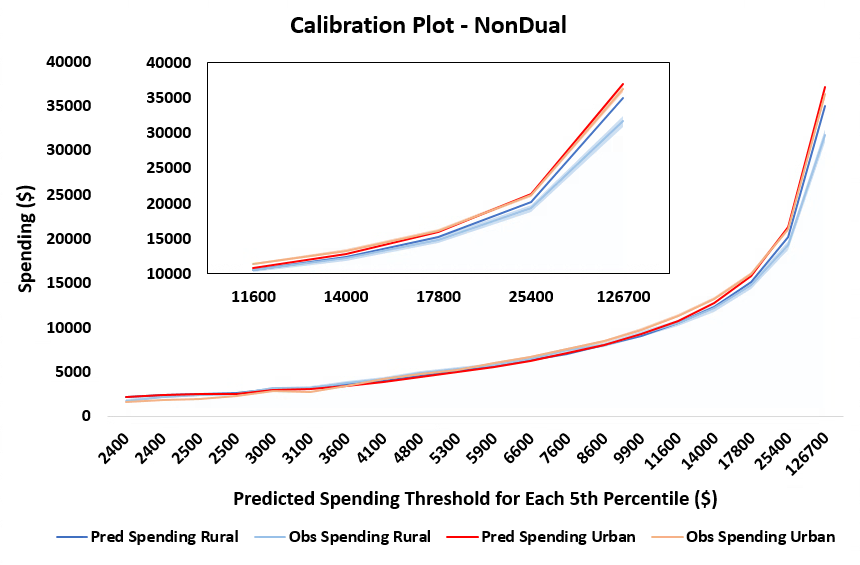


Supplemental Exhibit 2B


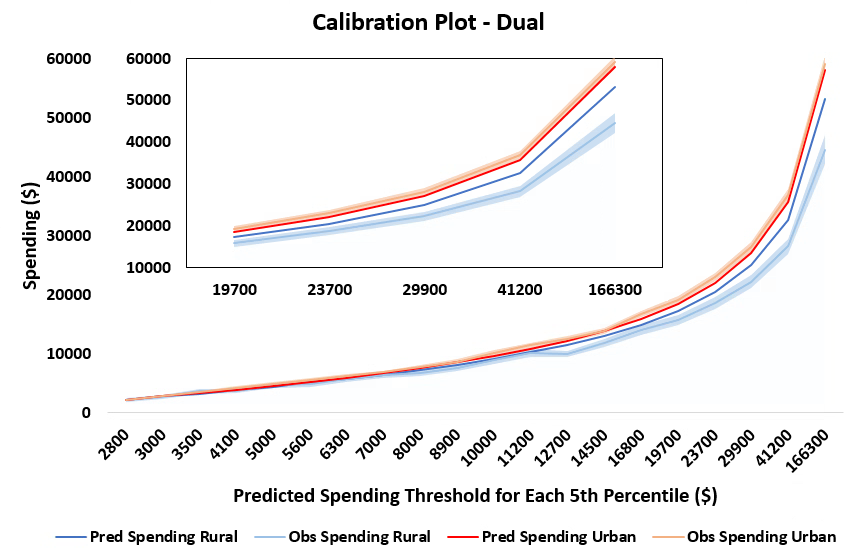


[Supplemental *Exhibit 2 represents predicted vs. observed spending for non-dual-eligible beneficiaries (Figure A) and dual-eligible beneficiaries (Figure B). To better visualize differences among high-spenders, insets visualize differences for the top 75^th^ percentile of the risk distribution. The x-axis represents predicted spending thresholds at every 5th percentile, obtained by binning the data into equal-sized groups and assigning the predicted spending amount at each percentile cutoff. The y-axis represents the mean spending amount in dollars within each bin. The 95% confidence intervals (CIs) are computed separately for predicted and observed values. For each bin, the mean and standard deviation (SD) are used to calculate the standard error, and the 95% confidence interval (CI) is defined as the mean ± 1.96 times the standard error.***Supplemental Exhibit 3. Calibration plot using our retrained HCC spending model**


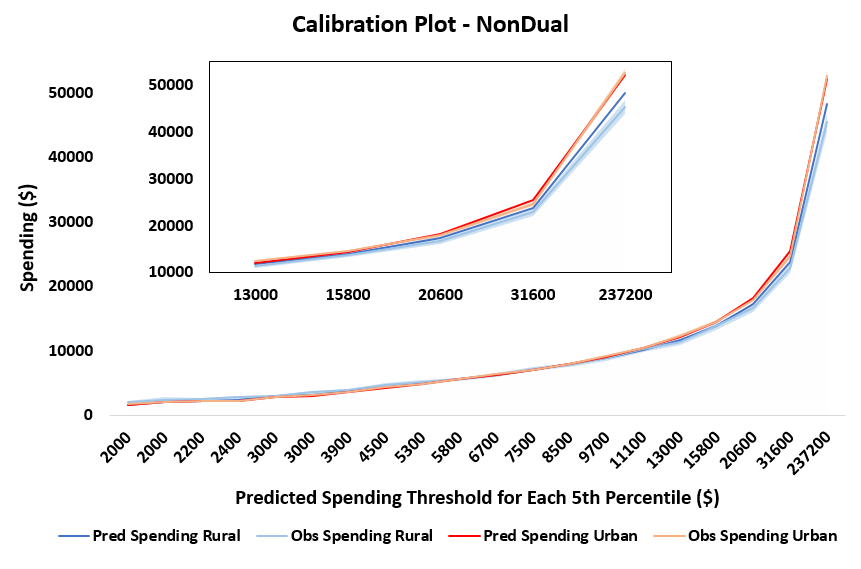


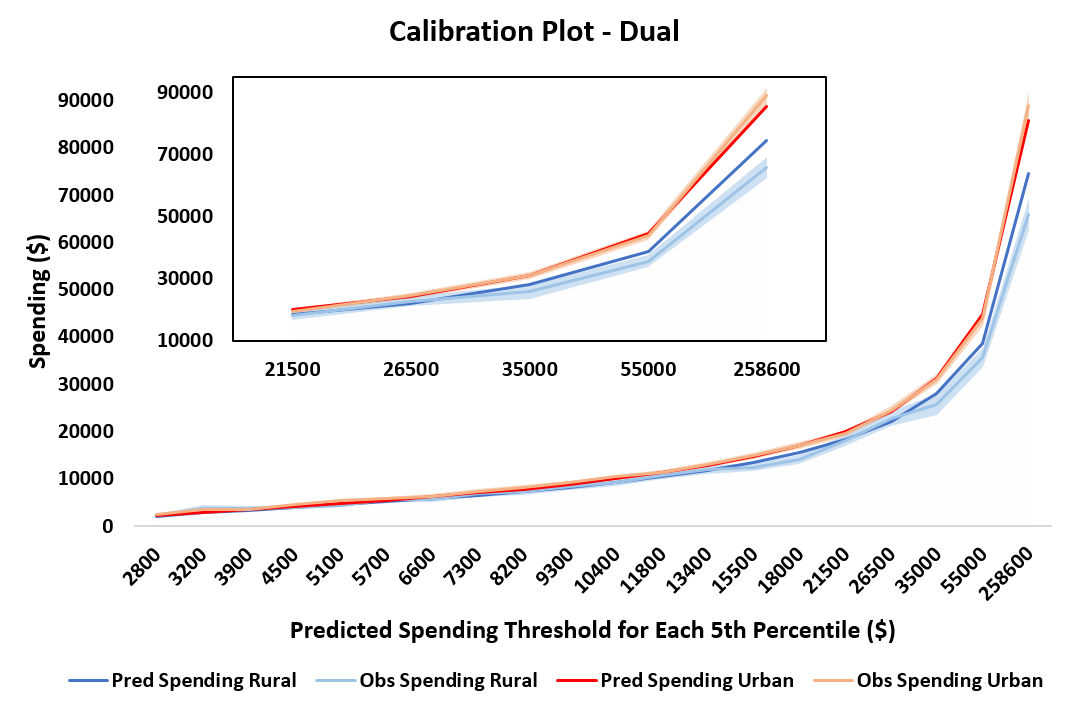


*Supplemental Exhibit 3 represents predicted vs. observed spending using the retrained model for non-dual-eligible beneficiaries (Figure A) and dual-eligible beneficiaries (Figure B). To better visualize differences among high-spenders, insets visualize differences for the top 75^th^ percentile of the risk distribution. The x-axis represents predicted spending thresholds at every 5th percentile, obtained by binning the data into equal-sized groups and assigning the predicted spending amount at each percentile cutoff. The y-axis represents the mean spending amount in dollars within each bin. The 95% confidence intervals (CIs) are computed separately for predicted and observed values. For each bin, the mean and standard deviation (SD) are used to calculate the standard error, and the 95% confidence interval (CI) is defined as the mean ± 1.96 times the standard error.*$SE=SD/\sqrt{n}$ $mean\pm1.96\times SE$

**Supplemental Exhibit 4. Predictive ratios of overall spending in retrained HCC Model.**


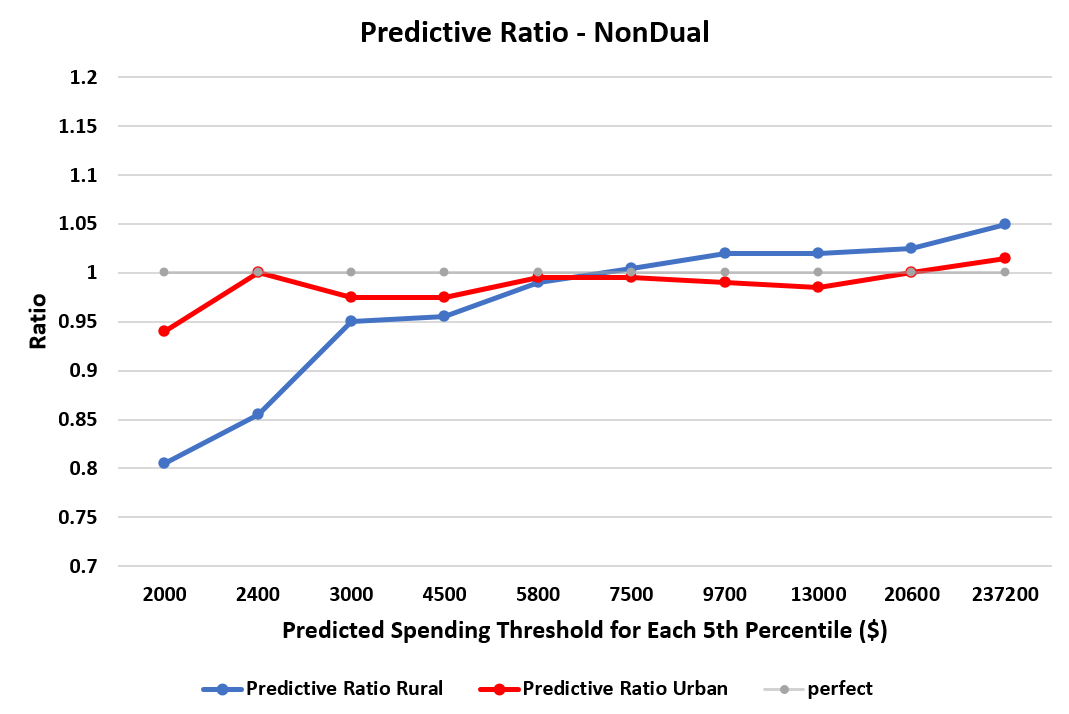


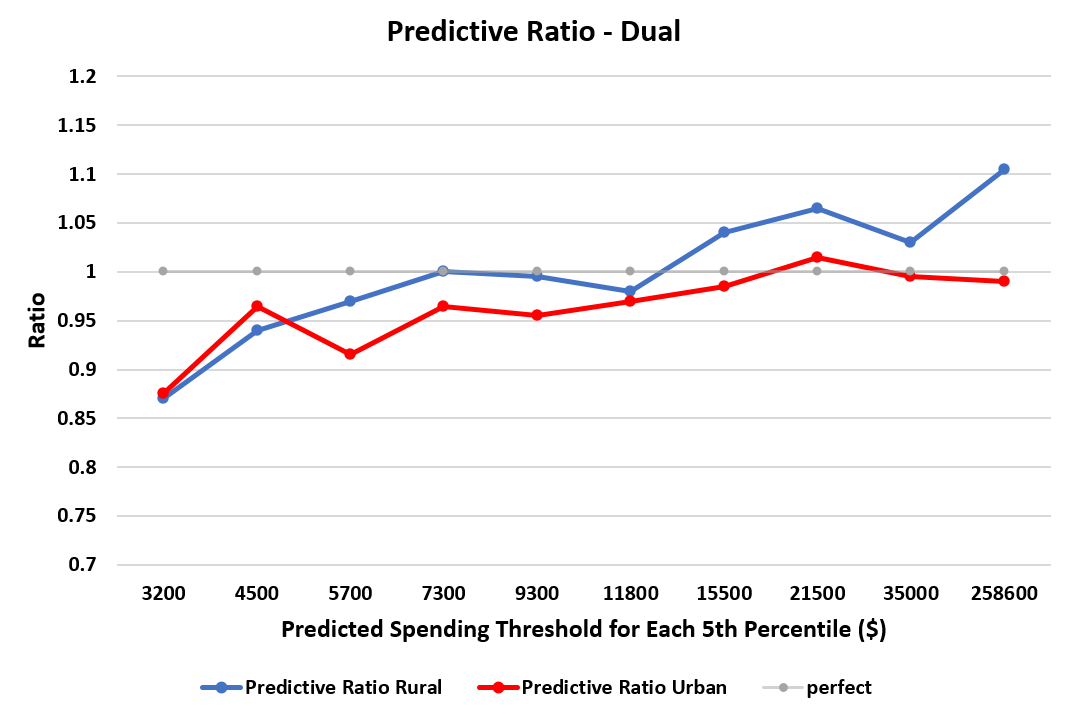


*Supplemental Exhibit 4 represents predictive ratios of overall spending using the retrained model for non-dual-eligible beneficiaries (Figure A) and dual-eligible beneficiaries (Figure B). The x-axis represents predicted spending thresholds at every 10^th^ percentile, obtained by binning the data into equal-sized groups and assigning the predicted spending amount at each percentile cutoff. The y-axis represents the predictive ratio, calculated as the mean predicted spending divided by the mean observed spending within each bin.*

**Supplemental Exhibits 5A-B. Excess mortality among non-dually insured and dually-insured beneficiaries.**


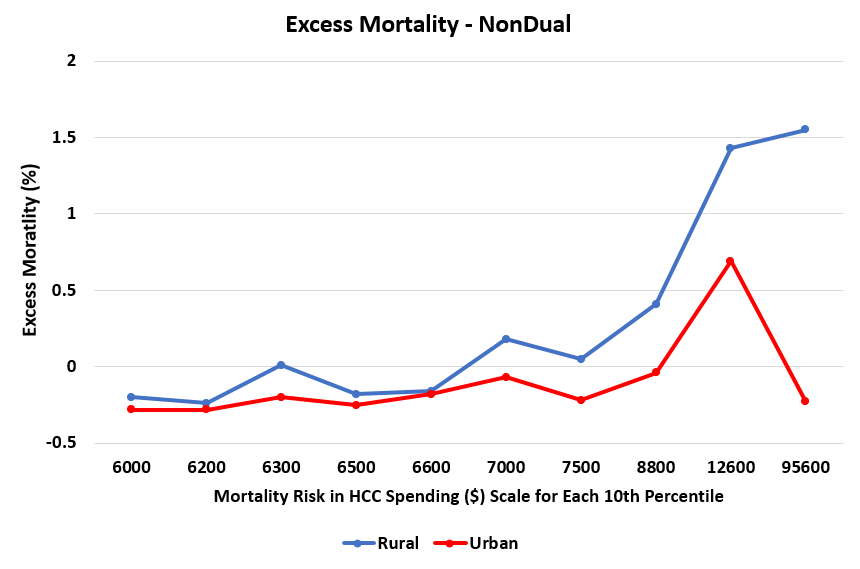


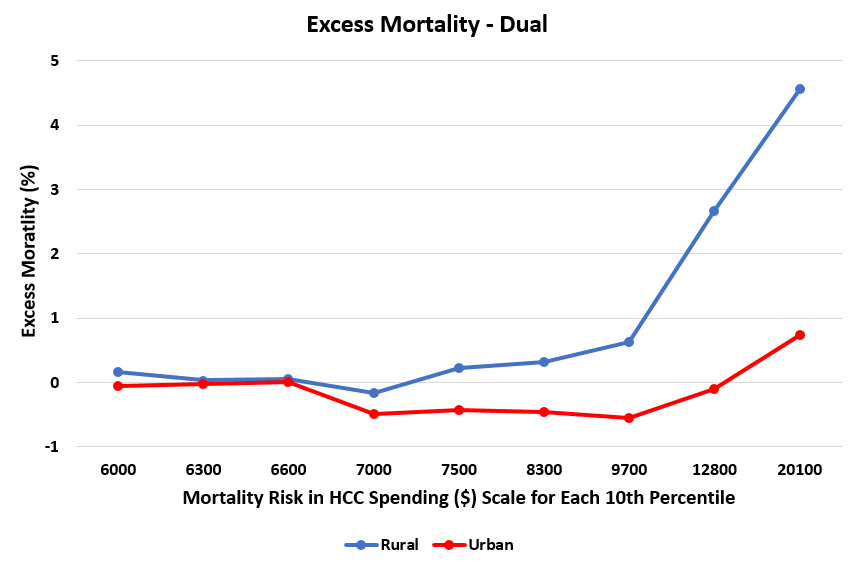


*Supplemental Exhibit 5 represents excess mortality for non-dual-eligible beneficiaries (Figure A) and dual-eligible beneficiaries (Figure B). The x-axis represents mortality probability thresholds at every 10^th^ percentile, transformed into the spending scale. The y-axis shows excess mortality, calculated as the difference between observed and predicted mortality within each bin.*

**Supplemental Exhibits 6A-E: Sensitivity analyses using alternative definition of rurality.**

**RUCC 7-9**

**HCC predicted spending vs observed annualized spending**
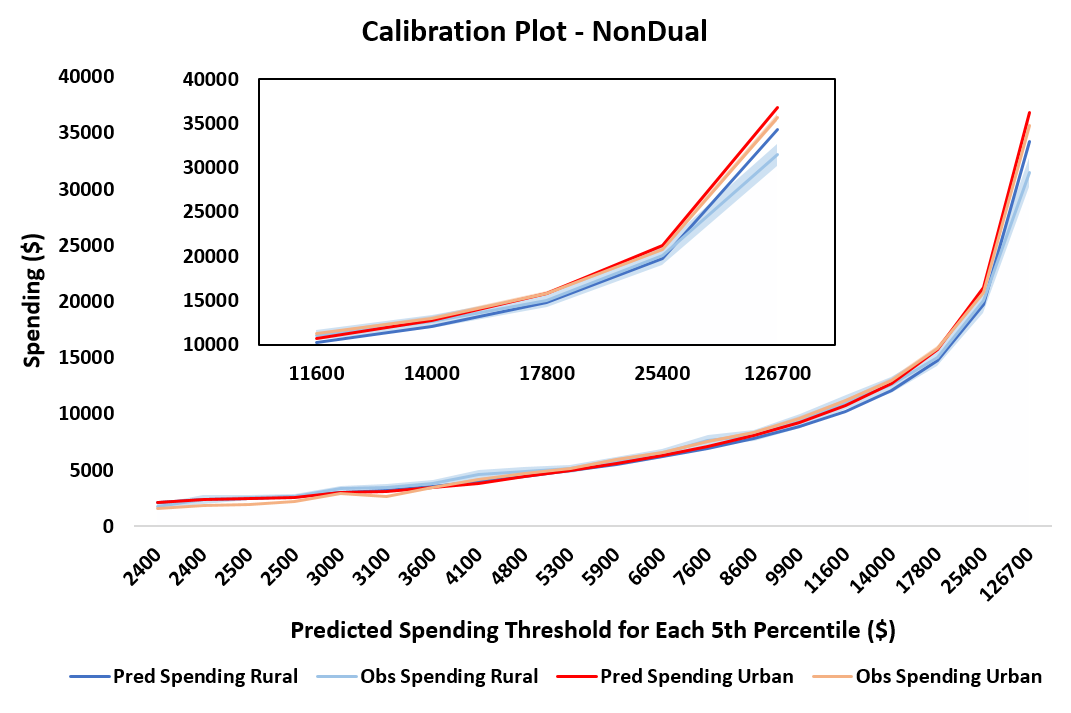


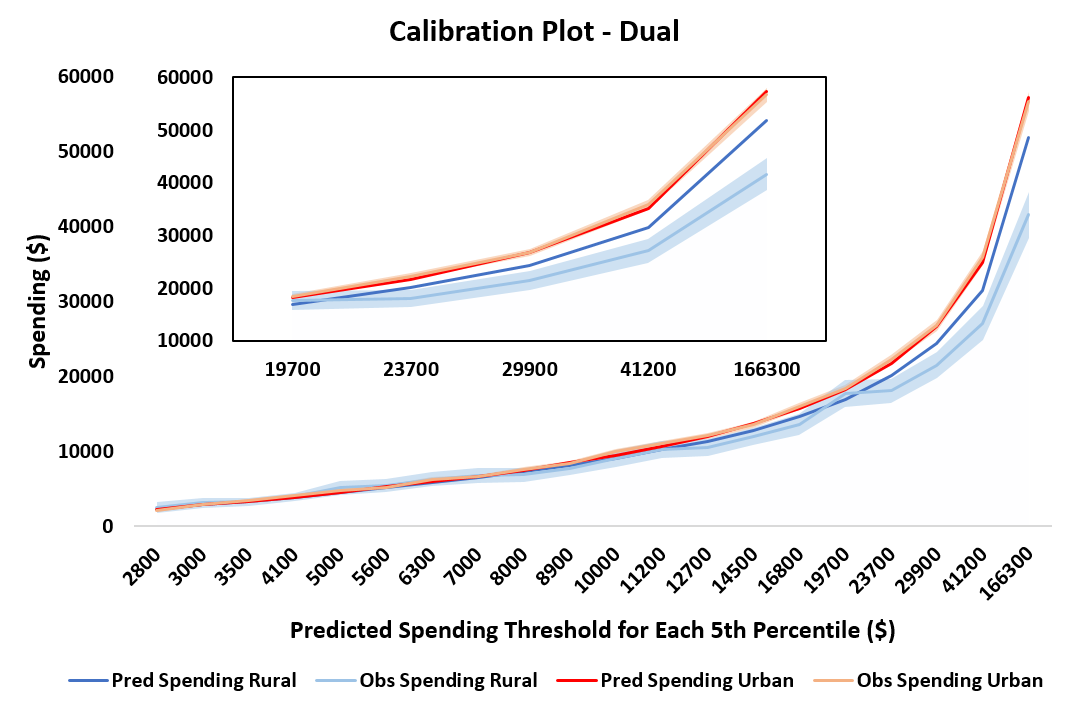


**Excess mortality among non-dually insured and dually-insured beneficiaries**


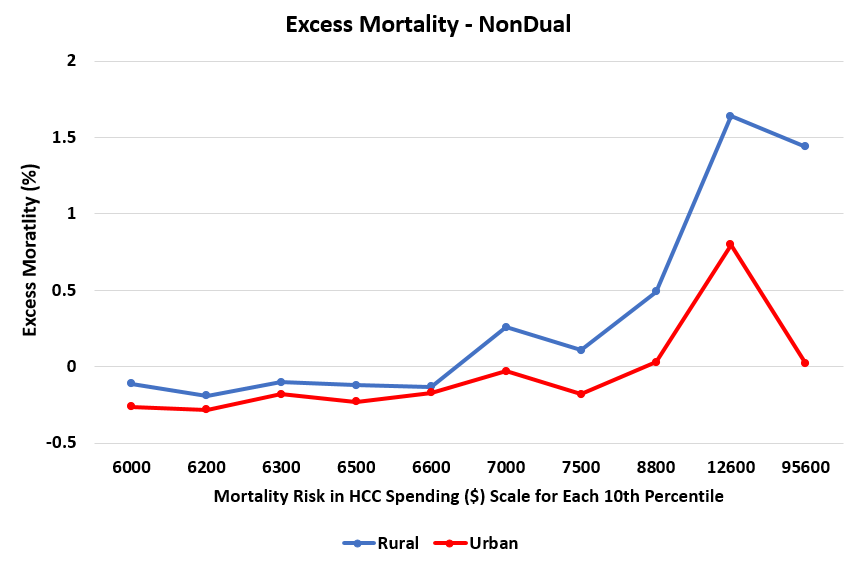


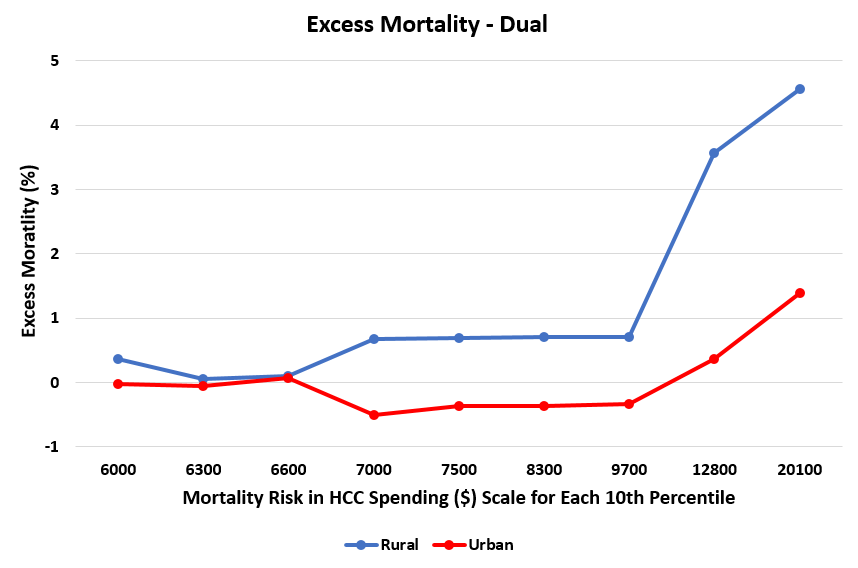


**CBSA**

**HCC predicted spending vs observed annualized spending**


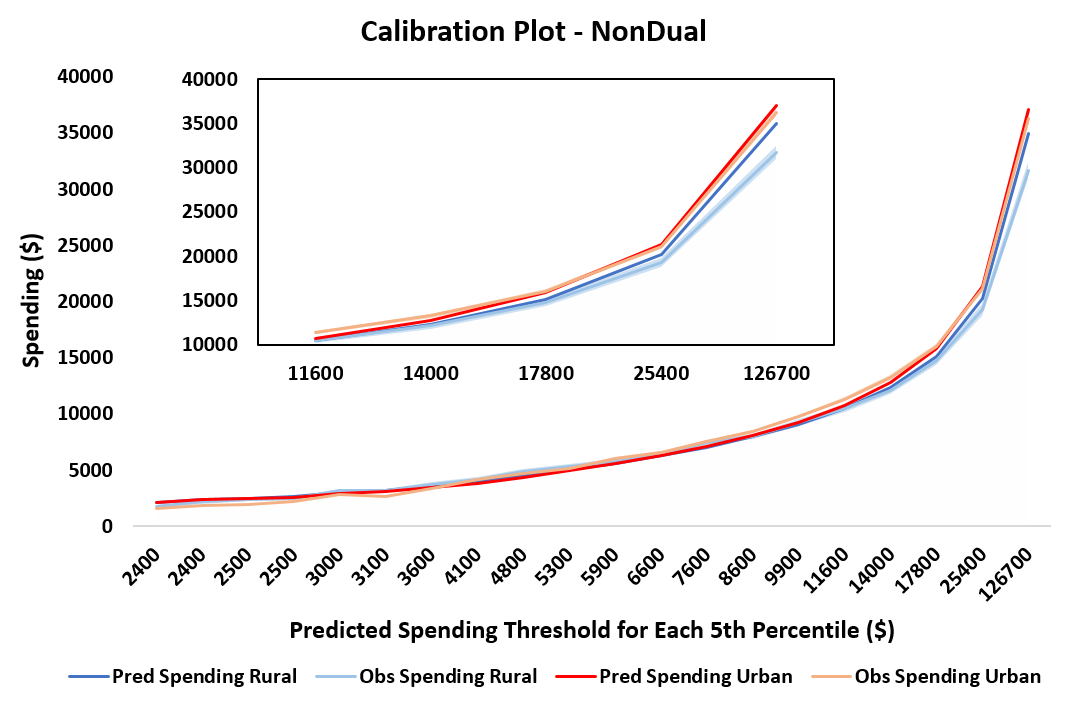


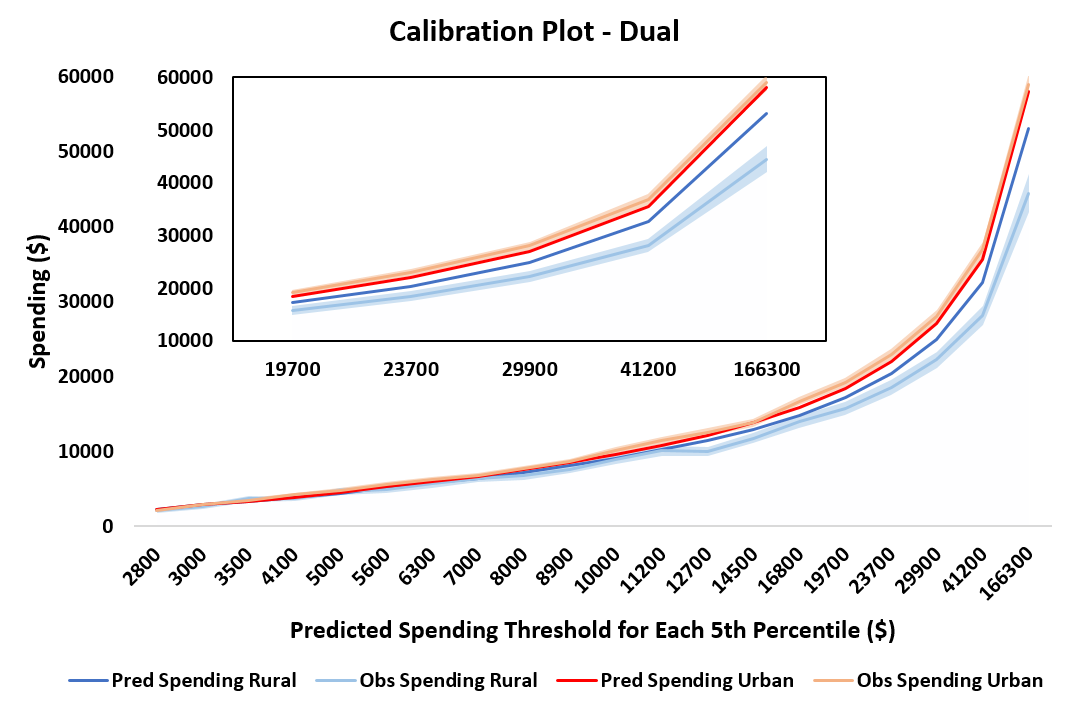


**Excess mortality among non-dually insured and dually-insured beneficiaries.**


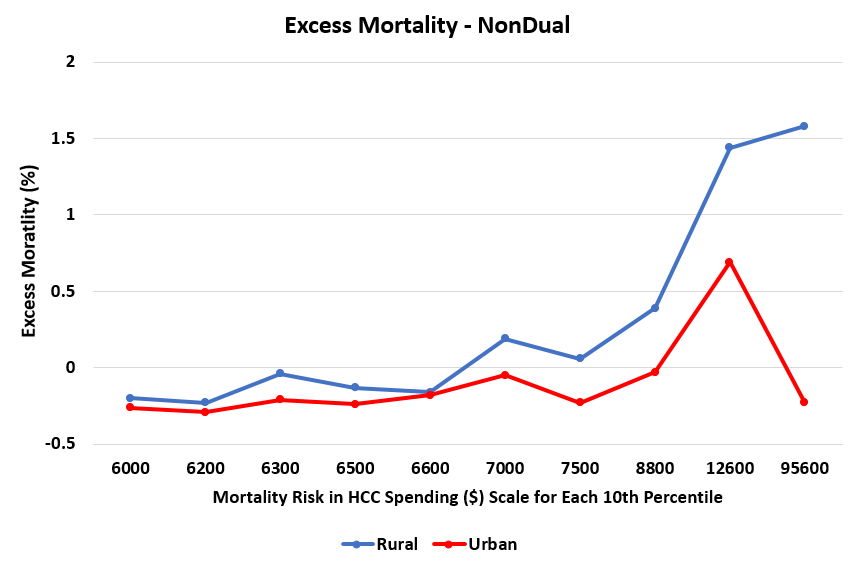


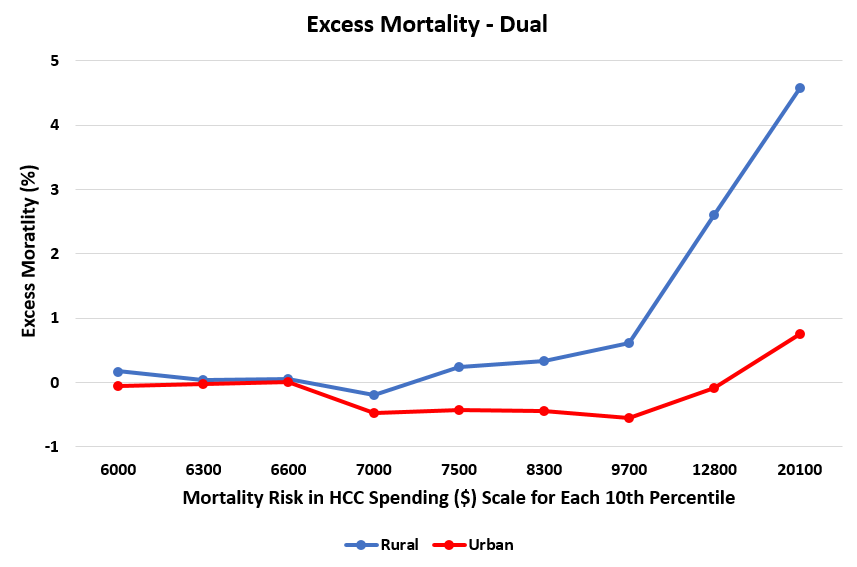


**Supplemental Exhibit 7A-B: Sensitivity analyses using standardized spending.**


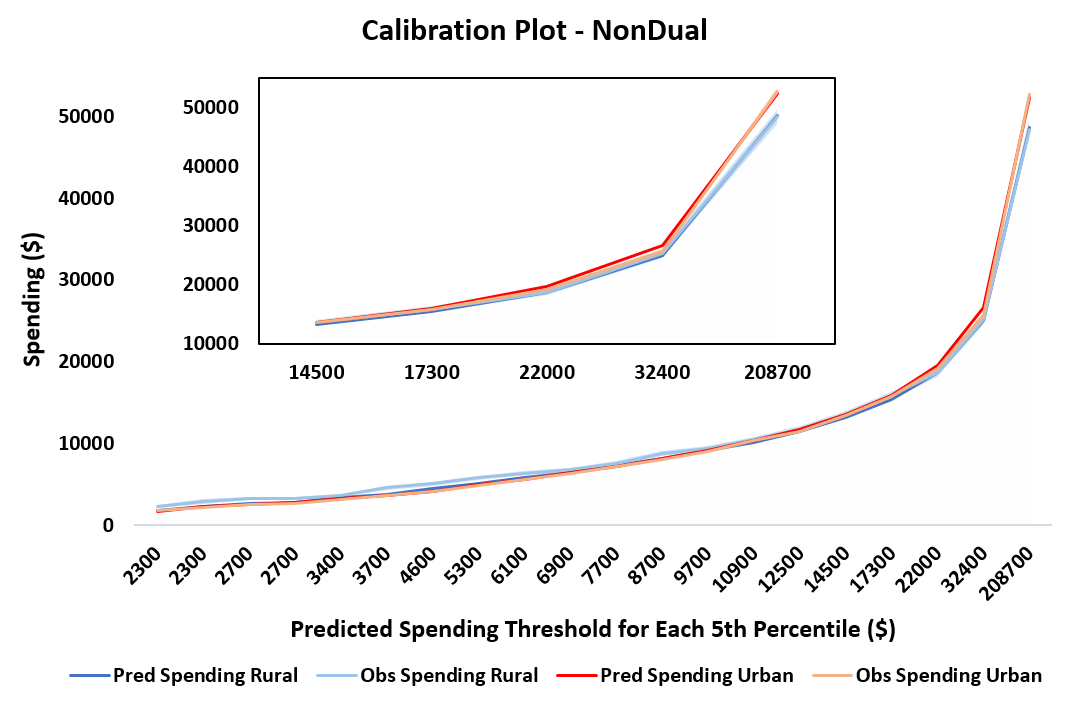


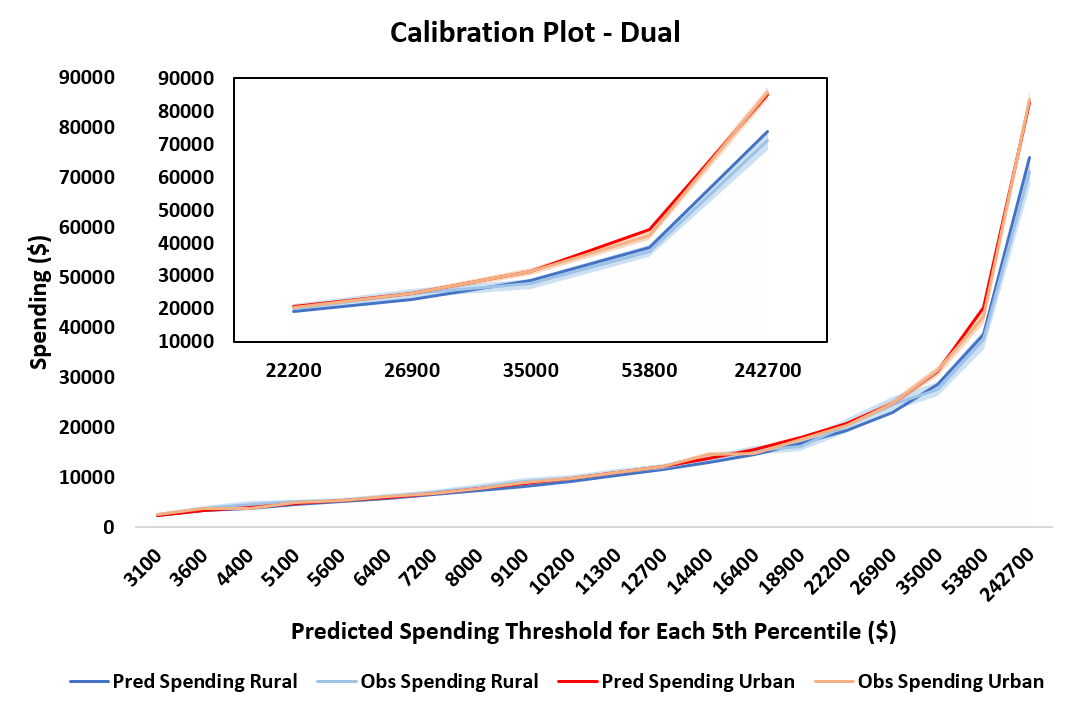


*Exhibit 7 represents predicted vs. observed spending using the retrained standardized spending model for non-dual-eligible beneficiaries (Figure A) and dual-eligible beneficiaries (Figure B). To better visualize differences among high-spenders, insets visualize differences for the top 75^th^ percentile of the risk distribution. The x-axis represents predicted standardized spending thresholds at every 5^th^ percentile, while the y-axis represents the mean standardized spending amount in dollars within each bin. The 95% confidence intervals (CIs) are computed separately for predicted and observed values. For each bin, the mean and standard deviation (SD) are used to estimate the standard error* $SE=SD/\sqrt{n}$*, with the 95% CI given by* $mean\pm1.96\times SE$*.*
